# Supplementary material for: STAT3‐dependent analysis reveals PDK4 as independent predictor of recurrence in prostate cancer
Source: Mol Syst Biol. 2020 Apr 23;16(4):e9247. doi: 10.15252/msb.20199247 (PMC7178451; doi:10.15252/msb.20199247)
Supplement: Supplementary file 1 — Appendix [file MSB-16-e9247-s001.pdf]

Appendix

Table of Contents

Appendix Figure S1: Clusters of the TCGA PRAD gene co-expression network..................2
Appendix Figure S2: Correlation of STAT3 target signatures with KEGG "OXPHOS"- and KEGG "Ribosome" signatures..3
Appendix Figure S3: Principal component analyses of proteomic data and protein-correlation in TMA...5
Appendix Figure S4: Association of *PDK4* with earlier death in primary PCa......................6
Appendix Figure S5: Association of *PDK4* with earlier biochemical recurrence or death in primary PCa...8
Appendix Figure S6: ENCODE STAT3 CHiP-seq data and STAT3 CHiP assays.................9
Appendix Supplementary Methods...11
References..12

Appendix Figure S1: Clusters of the TCGA PRAD gene co-expression network

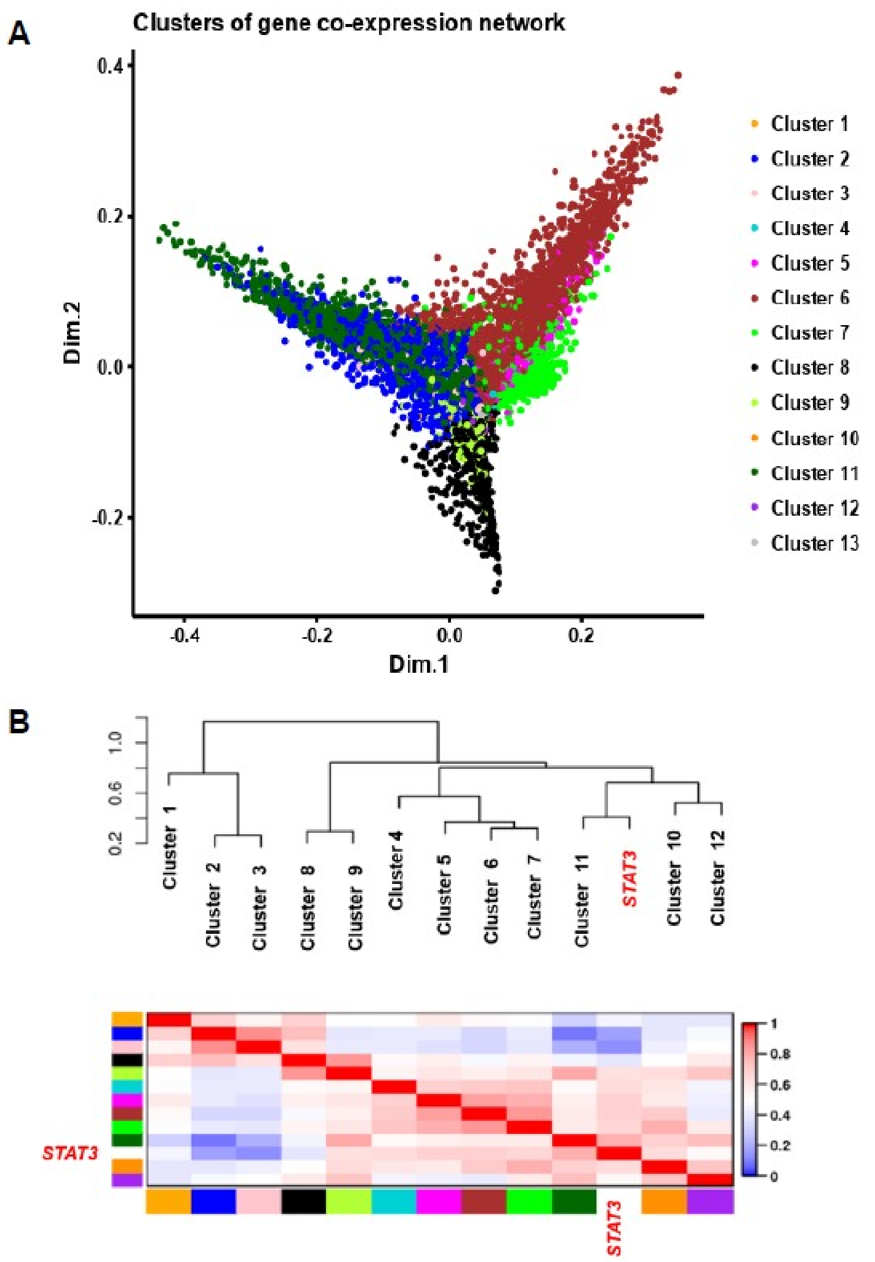

A. Multidimensional scaling (MDS) plot of the prostate cancer gene co-expression network. MDS plot was generated by using the topological overlap matrix (TOM). The topological overlap indicates, whether two genes share co-expression to a similar set of other genes. Colors represent different gene clusters. Genes in a cluster are interconnected by their high absolute correlation. The legend shows gene cluster numbers and respective assigned cluster colors. Smaller clusters may be occluded by larger ones in the MDS plot.

B. Graphical representation of the network of cluster eigengenes (= their first principal component). Dendrogram and heatmap plots show the relationships between the eigengenes and STAT3. Pearson- correlations between cluster eigengenes and the trait are indicated by a color bar ranging from red (1) to blue (-1).

Appendix Figure S2: Correlation of STAT3 target signatures with KEGG "OXPHOS"- and KEGG "Ribosome" signatures

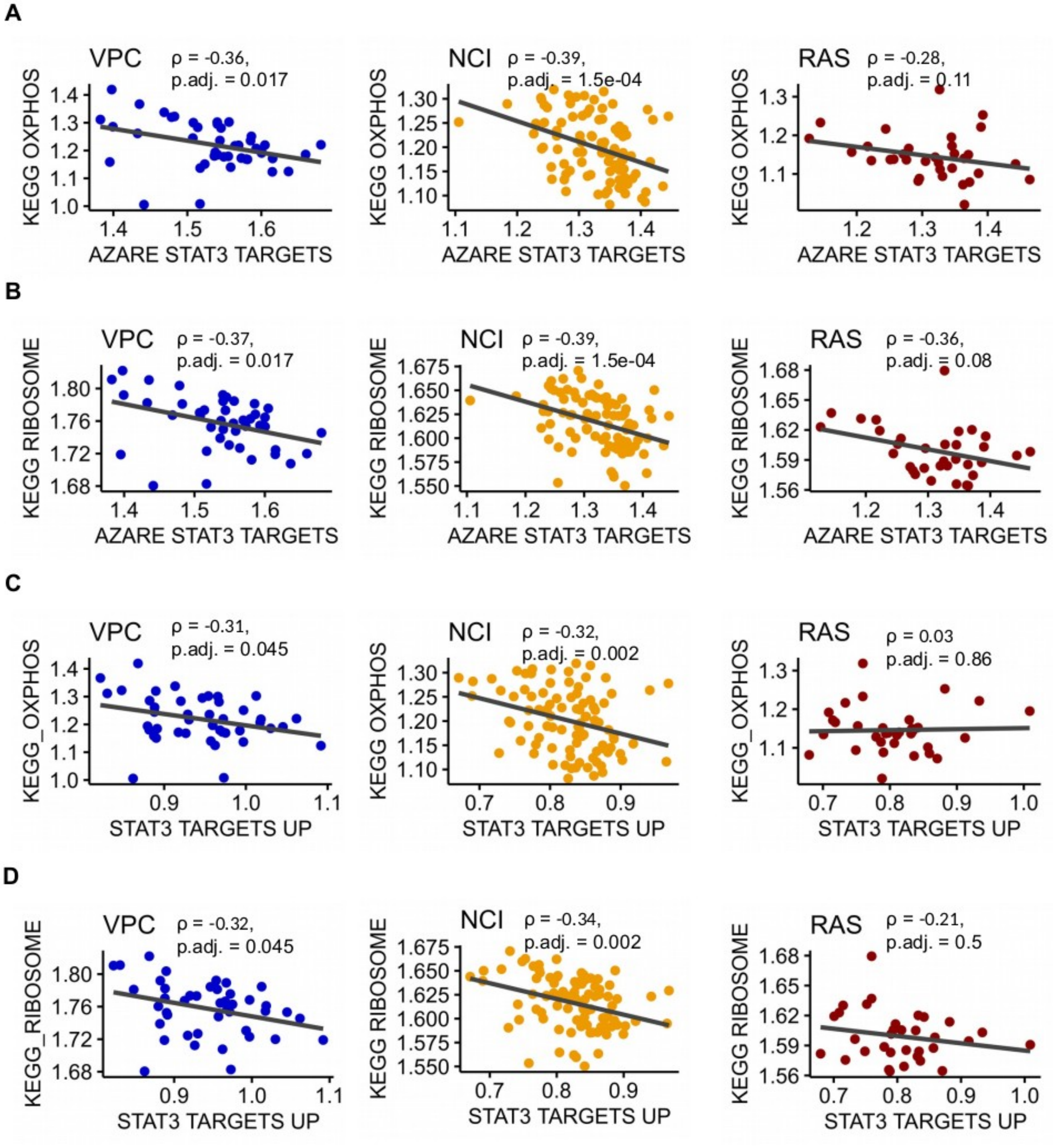

A. Pearson- correlation of "AZARE STAT3 TARGETS" - gene signatures with KEGG "OXPHOS"- gene signatures in three prostate cancer data sets. Gene signatures were assessed with ssGSEA. P-values were adjusted with Benjamini-Hochberg method. NCI = The Netherlands Cancer Institute, VPC = The Vancouver Prostate center, RAS = The Russian Academy of Science.

B. Pearson- correlation of "AZARE STAT3 TARGETS" - gene signatures with KEGG "Ribosome"- gene signatures in three prostate cancer data sets. Gene signatures were assessed with ssGSEA. P-values were adjusted with Benjamini-Hochberg method.

C. Pearson- correlation of "STAT3 TARGETS UP" - gene signatures with KEGG "OXPHOS"- gene signatures in three prostate cancer data sets. Gene signatures were assessed with ssGSEA. P-values were adjusted with Benjamini-Hochberg method.

D. Pearson- correlation of "STAT3 TARGETS UP" - gene signatures with KEGG "Ribosome"- gene signatures in three prostate cancer data sets. Gene signatures were assessed with ssGSEA. P-values were adjusted with Benjamini-Hochberg method.

Appendix Figure S3: Principal component analyses of proteomic data and protein-correlation in TMA

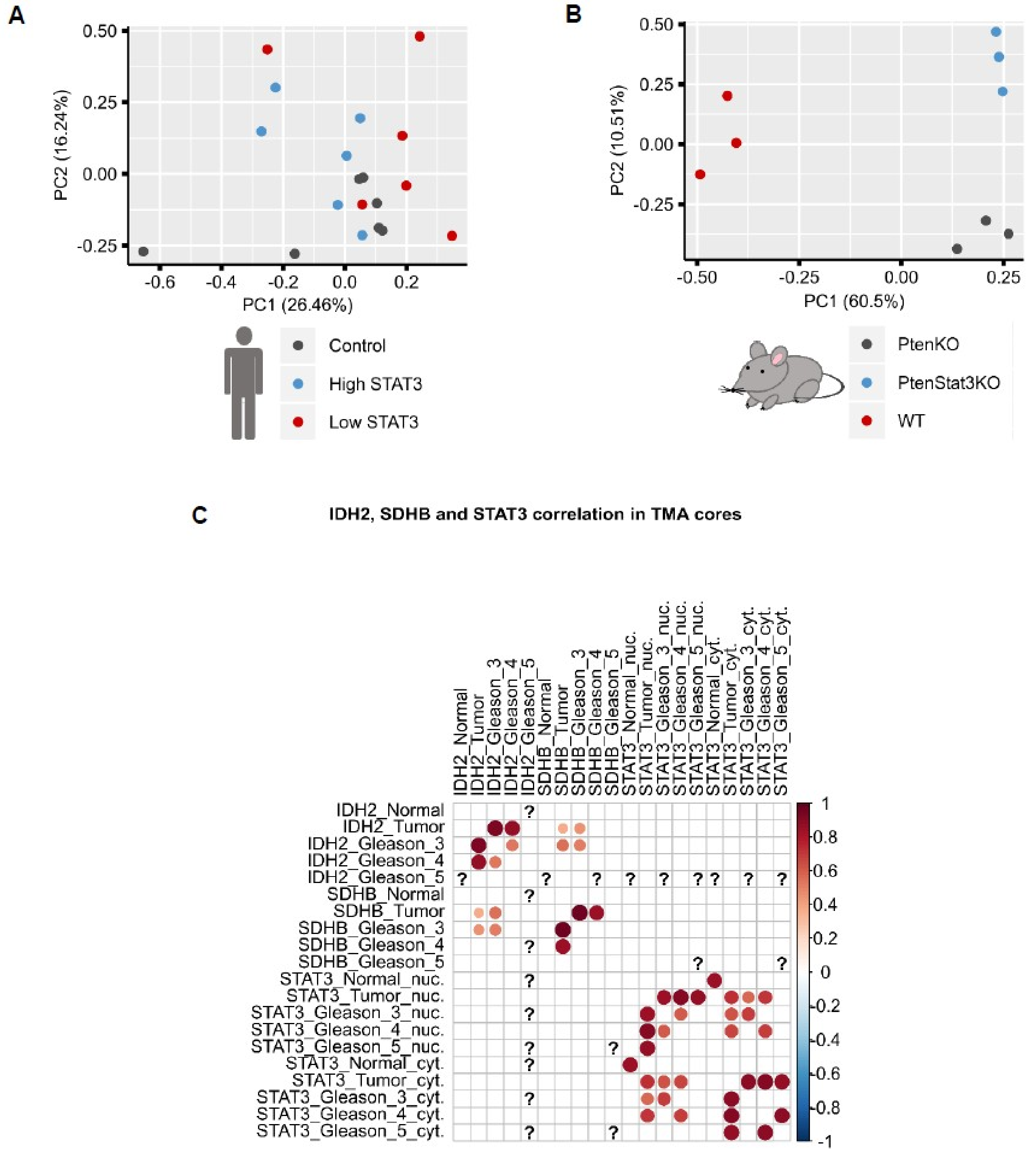

A - B. PCA of human (A) and murine (B) proteomic samples. Colors represent groups (Human: red = low STAT3, blue = high STAT3, grey = healthy prostate control; Mouse: red (PtenStat3Ko) = *PtenStat3*$^{pc-/-}$, blue (PtenKo) = *Pten*$^{pc-/-}$, grey = WT). PCA = Principal component analysis.

C. Spearman- correlations of IDH2, SDHB and STAT3 expression levels in human PCa TMA. Dot colors represent correlation (1 = red, -1 = blue), dot sizes represent adj. p-values ≤ 0.05. Only significant correlations are shown. Question marks indicate missing values (no paired samples available). P-values were adjusted with Benjamini-Hochberg method. Nuc = nuclear, cyt. = cytoplasmatic. TMA = Tissue Micro Array.

Appendix Figure S4: Association of *PDK4* with earlier death in primary PCa.

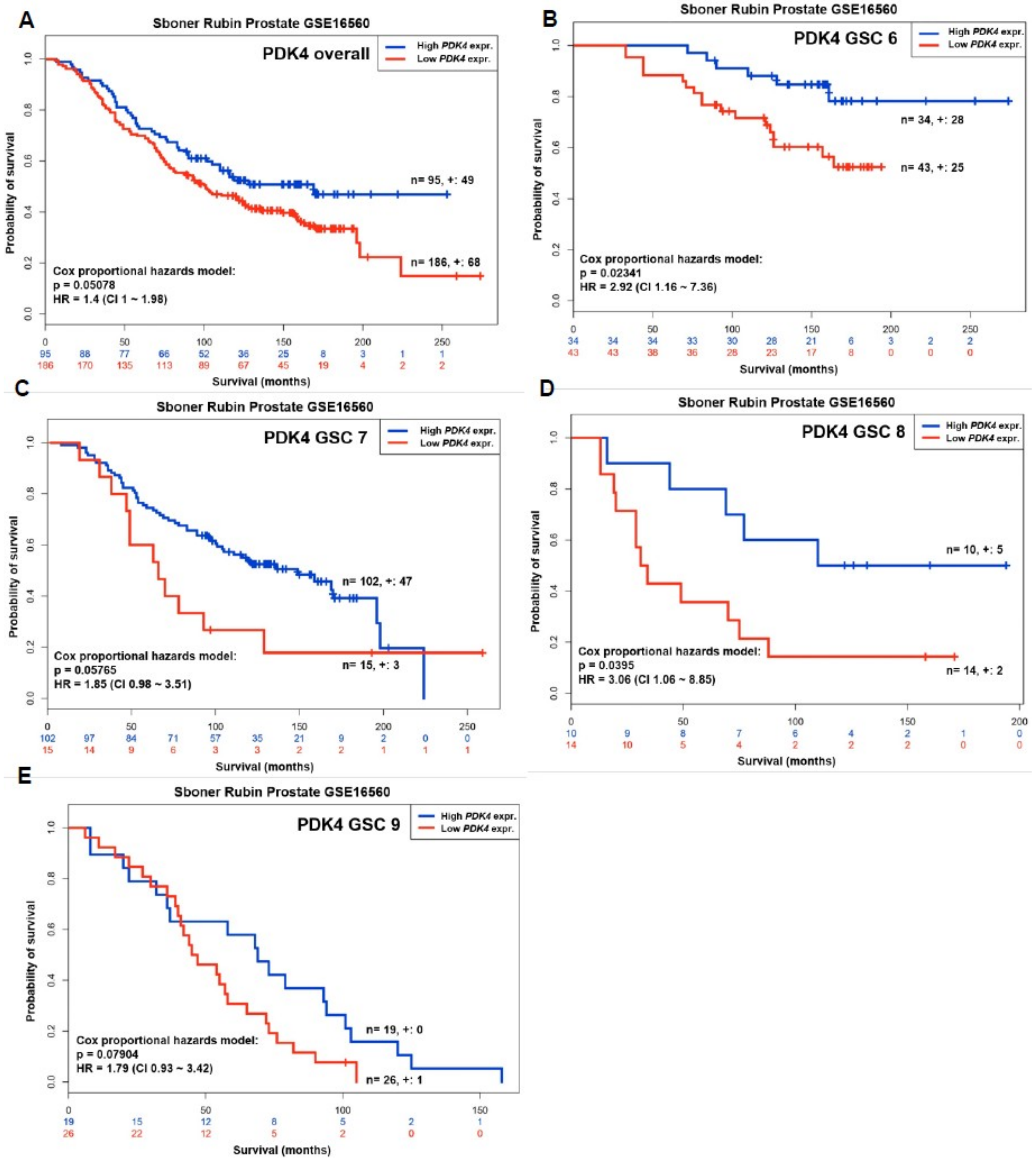

A. Kaplan-Meier plot showing survival time in months for *PDK4* expression groups over all GSCs in Sboner Rubin Prostate (GSE16560). Groups were generated by a maximizing split of samples after ranking by their prognostic index (risk score). Hazard ratio (HR), confidence intervals (CI) and p-value estimated by a Cox-model using groups as covariate are shown. Red = Low *PDK4* expression, blue = High *PDK4* expression, + = censored.

B - E. Kaplan-Meier plot showing survival time in months for *PDK4* expression groups for GSC 6 (B), 7 (C), 8 (D) and 9 (E) in Sboner Rubin Prostate (GSE16560). Groups were generated by a maximizing split of samples after ranking by their prognostic index (risk score). Hazard ratio (HR), confidence intervals (CI) and p-value estimated by a Cox-model using groups as covariate

are shown. GSC = Gleason Score. Red = Low *PDK4* expression, blue = High *PDK4* expression, + = censored.

Appendix Figure S5: Association of *PDK4* with earlier biochemical recurrence or death in primary PCa.

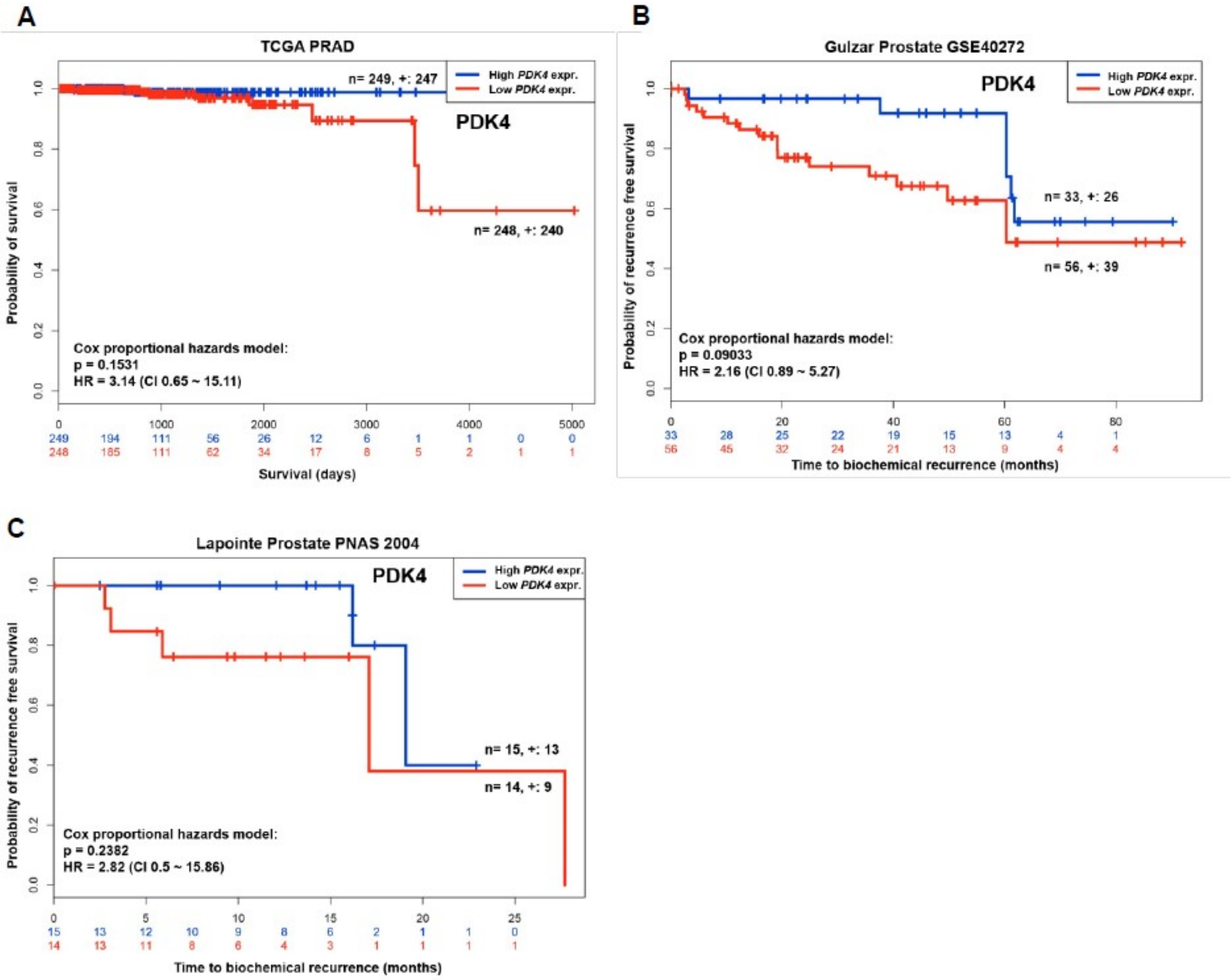

A. Kaplan-Meier plot showing survival time in days for *PDK4* expression groups in the TCGA PRAD dataset. Groups were generated by a median split of samples after ranking by their prognostic index (risk score). Hazard ratio (HR), confidence intervals (CI) and p-value estimated by a Cox-model using groups as covariate are shown. Red = Low *PDK4* expression, blue = High *PDK4* expression, + = censored.
B. Kaplan-Meier plots showing time to BCR in months for *PDK4* expression groups in the Gulzar Prostate dataset (GSE40272). Groups were generated by a maximizing split of samples after ranking by their prognostic index (risk score). Hazard ratio (HR), confidence intervals (CI) and p-value estimated by a Cox-model using groups as covariate are shown. Red = Low *PDK4* expression, blue = High *PDK4* expression, + = censored.
C. Kaplan-Meier plots showing time to BCR in months for *PDK4* expression groups in the Lapointe Prostate PNAS 2004 dataset. Groups were generated by a median split of samples after ranking by their prognostic index (risk score). Hazard ratio (HR), confidence intervals (CI) and p-value estimated by a Cox-model using groups as covariate are shown. Red = Low *PDK4* expression, blue = High *PDK4* expression, + = censored.

Appendix Figure S6: ENCODE STAT3 ChIP-seq data and STAT3 ChIP assays

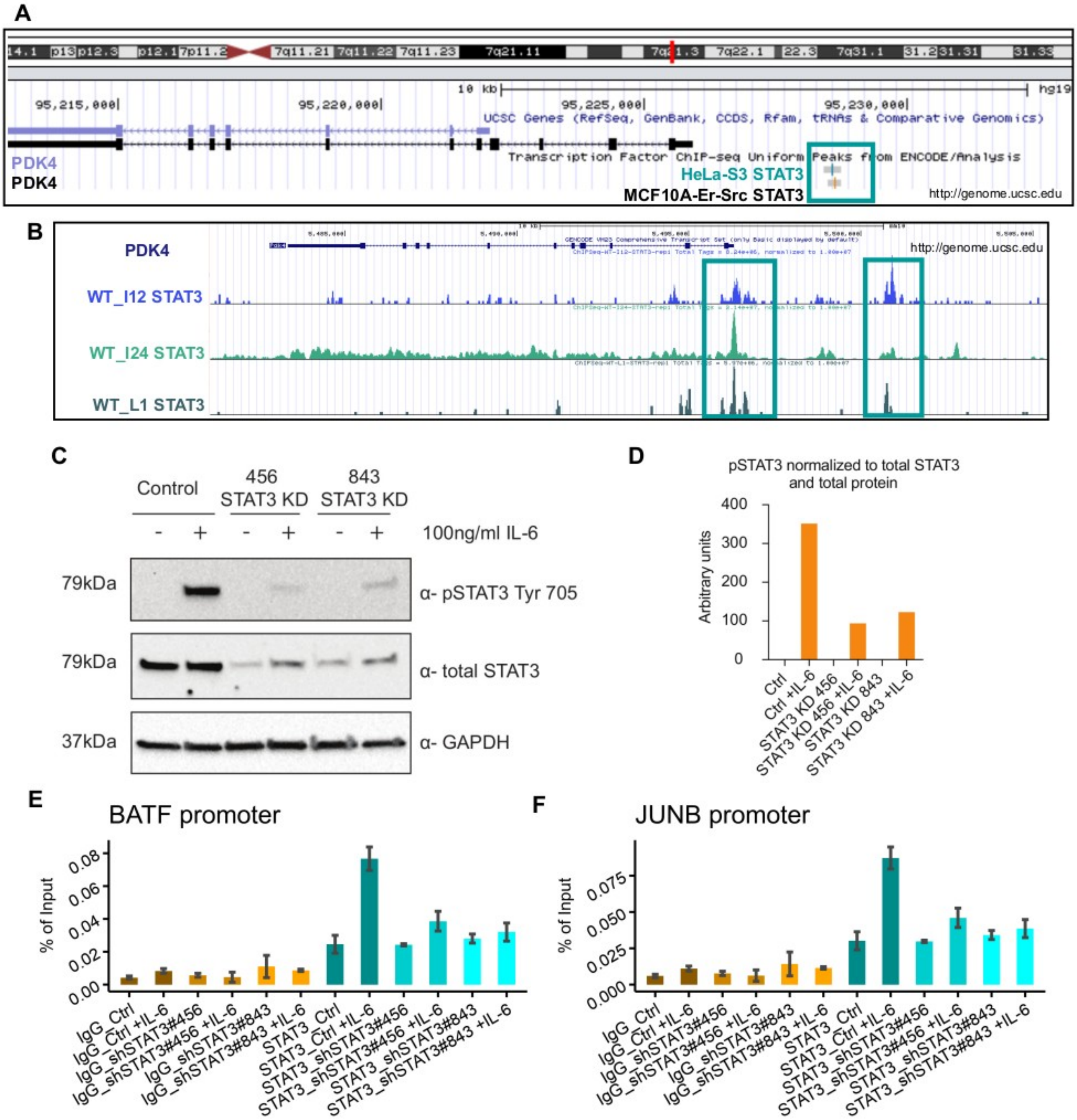

A. Genome Browser (http://genome.ucsc.edu) caption shows STAT3- ChIP-Seq uniform peaks from ENCODE Consortium in the promoter region of *PDK4* (hg19). HeLa-S3 STAT3 = ENCSR000EDC, GEO:GSM935276, MCF10A-Er-Src STAT3 = ENCSR000DOZ, GEO:GSM935457.

B. Genome Browser (http://genome.ucsc.edu) caption shows STAT3- ChIP-Seq peaks from mammary glands of wild type mice (GSE84115) in the promoter region of *PDK4* (mm10). WT = wild type mammary tissues, L1 = lactation day one, I12 = involution 12 hours, I24 = involution 24 hours.

C. Western blot of tyrosine- phosphorylated STAT3 (pSTAT3 Tyr 705), total STAT3 and GAPDH protein levels in 22Rv1 cells with or without stimulation with human IL-6. 456 STAT3 KD and 843 STAT3 KD = short hairpin knockdowns of *STAT3,* Control= scrambled control.

D. Quantification of tyrosine- phosphorylated STAT3 (pSTAT3) protein levels in 22Rv1 cells with or without stimulation with human IL-6 after western blotting. pSTAT3 signal was normalized to total protein lanes and total STAT3 expression. 456 STAT3 KD and 843 STAT3 KD = short hairpin knockdowns of *STAT*3, Ctrl= scrambled control, +IL-6 = IL-6 stimulated.

E.- F. ChIP assays from IL-6 stimulated or non-stimulated 22Rv1 cells with or without knockdown of STAT3 was immunoprecipitated with a STAT3 specific antibody (blue shades) and IgG antibody as negative control (orange shades) followed by qPCR with primer pairs for the *BATF* (E) and *JUNB* (F) promoter regions. Bars represent mean ± SD from 2 technical replicates. Precipitated DNA is presented as % of input. One representative experiment is shown. Ctrl = scrambled control, shSTAT3 = short hairpin knockdown of *STAT3,* +IL-6 = IL-6 stimulated.

Appendix Supplementary Methods

For the generation of PDK4 primers, a STAT3 peak in the promoter region of PDK4 from Encode STAT3 ChIP-seq HeLa-S3 data (ENCSR000EDC) was extracted with the UCSC Genome Browser (http://genome.ucsc.edu). Primer pairs were created with Primer3web v4.1.0 software (Untergasser *et al*, 2012; Koressaar & Remm, 2007; Kõressaar *et al*, 2018). The extracted STAT3 peak and selected primer pairs are listed below.

STAT3 peak from HeLa-S3 Encode Data

```
>hg19_wgEncodeRegTfbsClusteredV3_STAT3 range=chr7:95228402-95228784 5'pad=0
3'pad=0 strand=+ repeatMasking=none

GAAAGTCCCATGTCCCTAAGTACATTGATTGCCCAGCCTAGCTTACCCAG
CACTACTATTCTATGTGCTGTGGGCTGATTAAAGATCAGGTGAAATCATA
TGATGTTCACAGAGATGACCTAGGGCTAAATGTGAACTGAGGTTGCCAAA
ATTTTGTCTCTATGTCATCCCTTCTAATAATATTTATGAAGTTACTCTCA
CAGTGCATTGCATTCTTCTGTGTCATTTCTGTATGATGGGAACTGTTATT
ATAATTGGCAGCATTCATGATAGCTGGCCTAGAAAGGTCAGGTCAAGTTG
TTTACCCAGTTGGCTAAGATGCTATGTATTACCATGATTAGAGAGTGACC
TGGGAAAAGAGTGGCACTTCTCTTCTCAGGTTG
```

PDK4 5′upstream region / STAT3 peak reverse complement

```
>hg19_refGene_NM_002612 range=chr7:95214953-95228828 5'pad=0 3'pad=0 strand=-
repeatMasking=none

tgcttcacctctgtcccaactggatcacctccaagatttgtgttcaacct
gagaagagaagtgccactcttttcccaggtcactctctaatcatggtaat
acatagcatcttagccaactgggtaaacaacttgacctgacctttctagg
ccagctatcatgaatgctgccaattataataacagttcccatcatacaga
aatgacacagaagaatgcaatgcactgtgagagtaacttcataaatatta
ttagaagggatgacatagagacaaaattttggcaacctcagttcacattt
agccctaggtcatctctgtgaacatcatatgatttcacctgatctttaat
cagcccacagcacatagaatagtagtgctgggtaagctaggctgggcaat
caatgtacttagggacatgggactttcctaaattagacttgcctgtaatg
cataatttccattgcctttcctctgaaaatcagagtataattctttgtaa
tatatatttttgcttattccaggttccatatacagtgagaaaagcaaatg
catgctttacttttacttatgttcttaagacccaatctaagaaaaagaag
ggatttttaccttaaatttacttgcttcatcaaagagagtcttattcagt
tttttggctacttgaacacatttcgttgctgcatcaagctatgcaaatat
ttaataaaggattccaagtacaaaaatataaataatagtggcatcaaaac
ctgtctacaagtggagatgcaatttggaacttgcccatattcccacagct
gtttcagcttaagataagtgcttgagctgttgtgtttttggtttttttgt
ttttctgtcccactcaagtgcaactgctagagtctatacttggatcatcc
ttgaatattctctaagaacccaggccccatcctcttcattggaagatcat
aaatattgcatgtcttcacatagctgtttctcaaaggtctgaaaaaaata
tttggtcataacaggaagtctcaaattctcgaccatctataggtaatgta
tagacaatgggagaaatagtaggagagctaatatggtgcctccaaacagg
ccacataactgacacagggtttgccaggactgtttcagatccctcataat
ggtgttgattttagagaagcaaaattgatacctattagacatccaaatgg
agaaaccaaataggcaattgaataggagaatctggctcttggaagaaagt
ttgtgctagagatagaattggaaatctgtcctgtgatttaatgcagtctc
ctagacgaagagggcaaaggatgacagcagagcctaggatcactcatgac
tttcagagttgcccgttctgcttataaatgcagggcaggctgggcgcaat
ggctcactcctgtaatcccagcactatgggagaccgaggtgggtggatca
cttgaggtcaggagtttgagaccagcctggccaacatggagaaaccccat
cttcaccaaaaatagaaaaaattagctgagtgtggtggcgtgtgcctgtg
atcccagctactcagaagactgaggcagcagaatcgcttgaacctgggag
gccgaggttgcagtgagccgagatggcaccactgcactccagcctgggag
acagtgtgactctgtctcaataaataaagtaattaattaattaattaaaa
```

PDK4 promoter primer pair 2

PDK4 promoter primer pair 1

```
taaaataaacgcagggggaggaggccaaaaaggtttttataacaaacta
tcatttttaacacctcataaattcccattctattacatttgtaaccaact
ctcttactcccaactttcccaaacacggtggcttcattccctagtccttt
tagagtgtcgggagaggaaaaggaggttagtatgacagggtaatgtgtct
caaccactgtcattaatttaataaatattgagtagtgaaaaagtaggaat
cacaaagctgcatcagacagggtccctaccctgatctctttcaggaaagg
ggggagtcagagagggataatataaaaacataaatagctatagaatagta
ccatgaaagaccaaggccttatttggaagaagtgaggagaagtaatcaag
gaagacctttgcagcagacagcttgtgccagacatttgagggtagaatg
gaatgtcttaggtaaagatttggagtaaggacatttcgtacagggcagag
tgaacaaattctctgatcaccgcaaaaggtaaggcaaactgacaaattga
tcccttttttaattttgtttttaattaacagaactaaacctaacggaaaga
atgtgttgccggagaagaacgaattgatggccttttattaccacaggaaa
aagtaaatgtatctgggcagcccctcaagtctcagcaaaaggaacgtaca
gttcctgccatgtgccggtctcttctgaagaaaagggggcggtgggggtg
ggggcgcaggtctttaggttgtttattcctttctctctgtccagtggcag
agaagcccatacttttgaacgcagcttgttggctactgtaaaagccctgc
tctgagcaaggaccaatgagcacgcggagtccaaactcttggaaacagtt
tgtggccaggagtacttgacattgagacagcctccgagttgtaaacaagg
gcgagcctgggcgggaccccagcccacgcgactcggagccccgtcccaag
aggctaatcttaagcccacgttgccccagatacctgtttctgcttcctct
tccctgttcttcccacccttttttccgtcacagccgcgggcaccggtgcca
gggcactccgtggtcaccgtgccaggccattctgtgatgaccgcggctgg
aggggcggagcccatagttctttctctgatctgattggcgcgacctggag
ttcaggacgcgtttccaagttccagtgactcctcctgtttgggactcggg
gggagagtgcggggagacaaataaaacctcgggcggcggcggctggtggg
AAGACTTGAACTTGAATCTCGAACCACTGCATCTCCGACTCTGCCCAGAC
TCTTCACTCCGCGGCACCCTCAAACCCCAGCCCAGGCCGGGGCGCACAAG
CCAGCCAGCGCACCTGCAGTCCTCGCCCGGACGCGCCGCGCCCCCTCGGA
ACCAGGCTCTGCTCCGAGCAGCCTTCGCCCCTCAAGCCAGCCACAGTCCC
CGCCAGGCCGGGTGGGCGTCAAGATGAAGGCGGCCCGCTTCGTGCTGCGC  Translational START site
AGCGCTGGCTCGCTCAACGGCGCCGGCCTGGTGCCCCGAGAGGTGGAGCA
TTTCTCGCGCTACAGCCCGTCCCCGCTGTCCATGAAGCAGCTACTGGACT
TTG// Exon 1 end
```

References

Kõressaar T, Lepamets M, Kaplinski L, Raime K, Andreson R & Remm M (2018) Primer3_masker: integrating masking of template sequence with primer design software. *Bioinformatics* **34:** 1937–1938

Koressaar T & Remm M (2007) Enhancements and modifications of primer design program Primer3. *Bioinformatics* **23:** 1289–1291

Untergasser A, Cutcutache I, Koressaar T, Ye J, Faircloth BC, Remm M & Rozen SG (2012) Primer3—new capabilities and interfaces. *Nucleic Acids Res* **40:** e115
