# Supplementary material for: STAT3‐dependent analysis reveals PDK4 as independent predictor of recurrence in prostate cancer
Source: Mol Syst Biol. 2020 Apr 23;16(4):e9247. doi: 10.15252/msb.20199247 (PMC7178451; doi:10.15252/msb.20199247)
Supplement: Supplementary file 2 — Expanded View Figures PDF [file MSB-16-e9247-s002.pdf]

Expanded View Figures

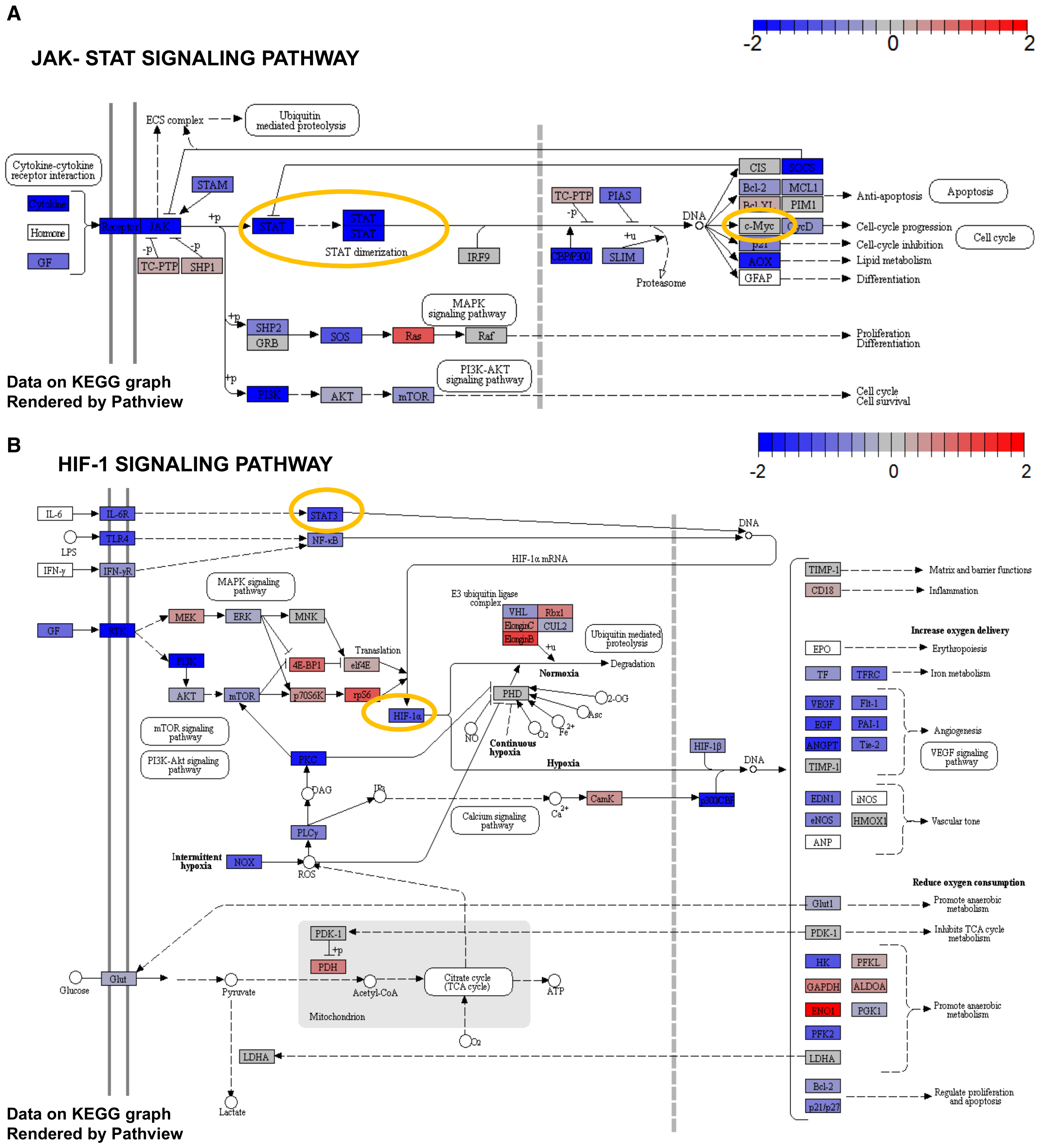

Figure EV1.

 © 2020 The Authors

Figure EV1. Scheme of down-regulated KEGG pathways in low STAT3 TCGA samples.

A KEGG representation of the JAK-STAT signaling pathway in low STAT3 versus high STAT3 TCGA samples after testing for signaling KEGG pathways. *STAT3* and *c-Myc* are encircled in yellow. Color bar indicates *z*-scored deregulation of genes. Blue, down-regulation; red, up-regulation.

B KEGG representation of the HIF-1 signaling pathway in low STAT3 versus high STAT3 TCGA samples after testing for signaling KEGG pathways. *STAT3* and *HIF-1α* are encircled in yellow. Color bar indicates *z*-scored deregulation of genes. Blue, down-regulation; red, up-regulation.

© 2020 The Authors

A

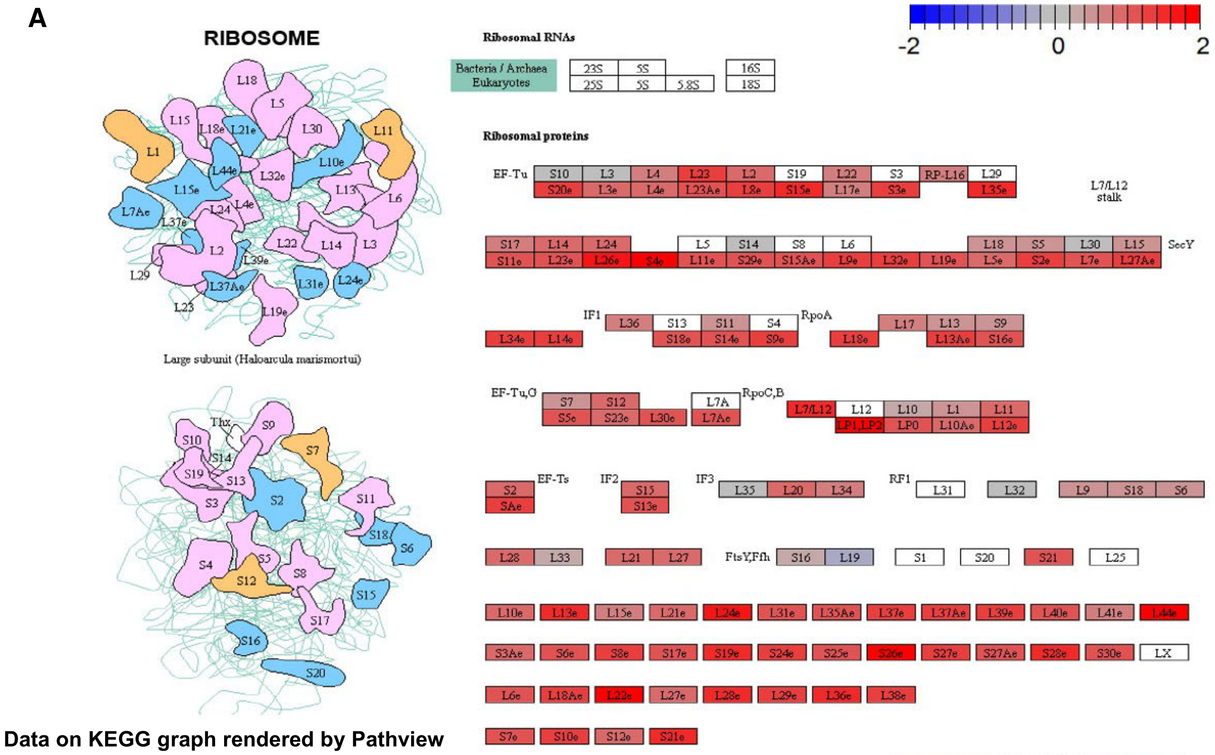

B

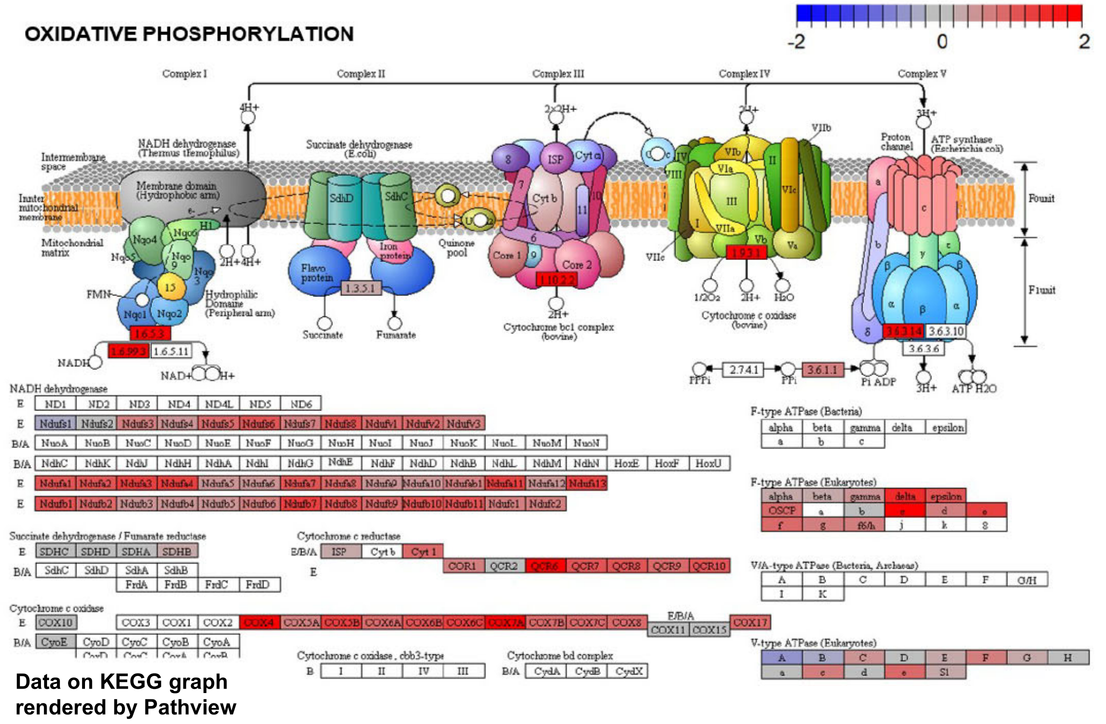

Figure EV2. Scheme of up-regulated KEGG pathways in low STAT3 TCGA samples.

A KEGG representation of the ribosome in low STAT3 versus high STAT3 TCGA samples after testing for signaling KEGG pathways. Color bar indicates *z*-scored deregulation of genes. Blue, down-regulation; red, up-regulation.

B KEGG representation of oxidative phosphorylation in low STAT3 versus high STAT3 TCGA samples after testing for metabolic KEGG pathways. Color bar indicates *z*-scored deregulation of genes. Blue, down-regulation; red, up-regulation.

 © 2020 The Authors

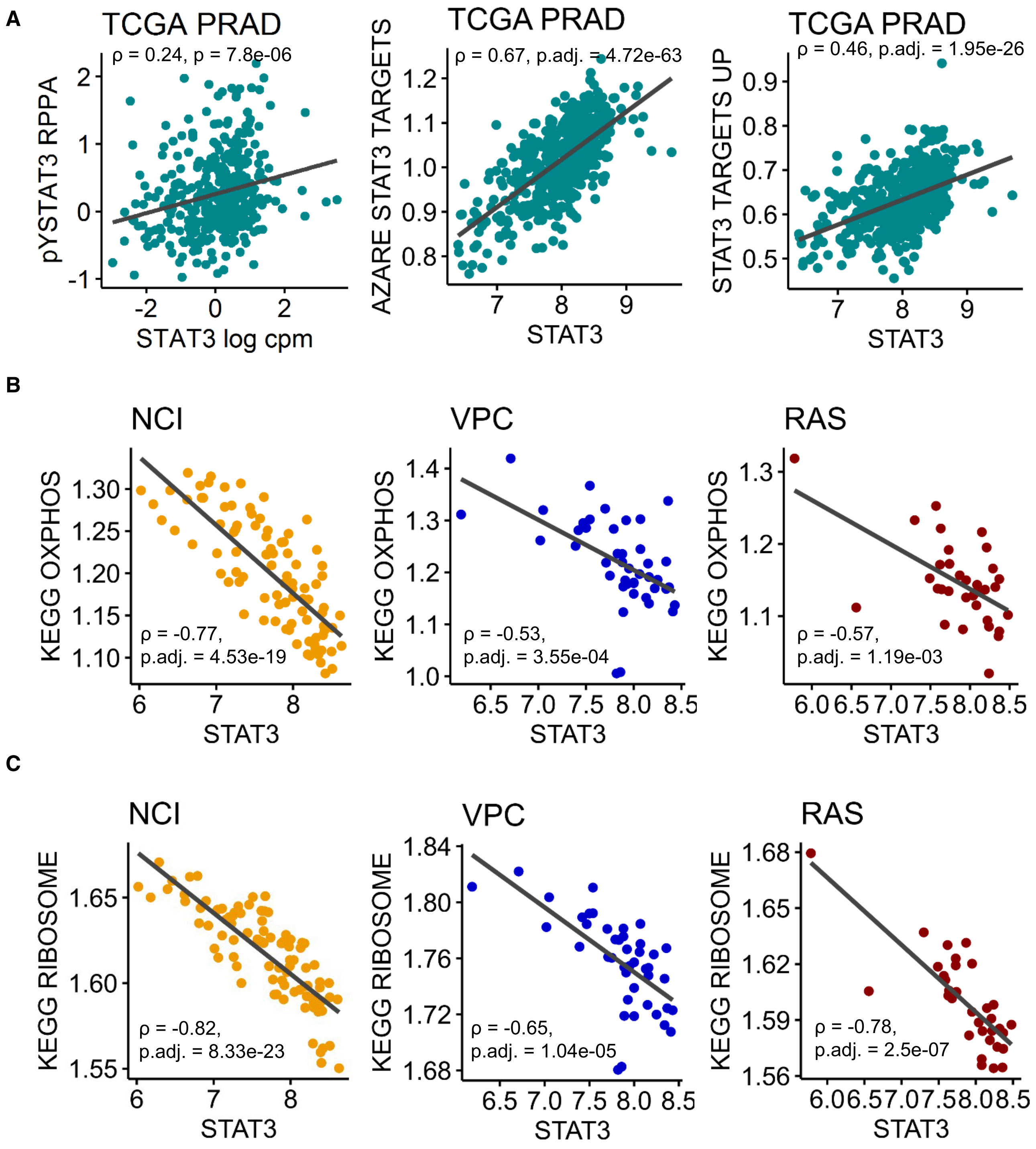
A
TCGA PRAD
ρ = 0.24, p = 7.8e-06
pYSTAT3 RPPA
STAT3 log cpm
TCGA PRAD
ρ = 0.67, p.adj. = 4.72e-63
AZARE STAT3 TARGETS
STAT3
TCGA PRAD
ρ = 0.46, p.adj. = 1.95e-26
STAT3 TARGETS UP
STAT3
B
NCI
KEGG OXPHOS
ρ = -0.77,
p.adj. = 4.53e-19
STAT3
VPC
KEGG OXPHOS
ρ = -0.53,
p.adj. = 3.55e-04
STAT3
RAS
KEGG OXPHOS
ρ = -0.57,
p.adj. = 1.19e-03
STAT3
C
NCI
KEGG RIBOSOME
ρ = -0.82,
p.adj. = 8.33e-23
STAT3
VPC
KEGG RIBOSOME
ρ = -0.65,
p.adj. = 1.04e-05
STAT3
RAS
KEGG RIBOSOME
ρ = -0.78,
p.adj. = 2.5e-07
STAT3

Figure EV3.

Figure EV3. Correlation of *STAT3* with STAT3 target, ribosome, and OXPHOS signatures.

A Pearson correlation of *STAT3* log counts per million (cpm) with tyrosine-phosphorylated (pY) STAT3 Reverse Phase Protein Array (RPPA) protein levels (left, *z*-scored), "AZARE STAT3 TARGETS" (middle), and "STAT3 TARGETS UP" gene signatures (right) in TCGA PRAD. Gene signatures were assessed with ssGSEA. *P*-values were adjusted with Benjamini–Hochberg method. *P*. adj., adjusted *P*-value.

B Pearson correlation of *STAT3* log cpm with KEGG "OXPHOS" gene signature in three prostate cancer data sets. Gene signatures were assessed with ssGSEA. *P*-values were adjusted with Benjamini–Hochberg method. NCI, The Netherlands Cancer Institute; VPC, The Vancouver Prostate Center; RAS, The Russian Academy of Science.

C Pearson correlation of *STAT3* log cpm with KEGG "Ribosome" gene signature in three prostate cancer data sets. Gene signatures were assessed with ssGSEA. *P*-values were adjusted with Benjamini–Hochberg method.

Source data are available online for this figure.

 © 2020 The Authors

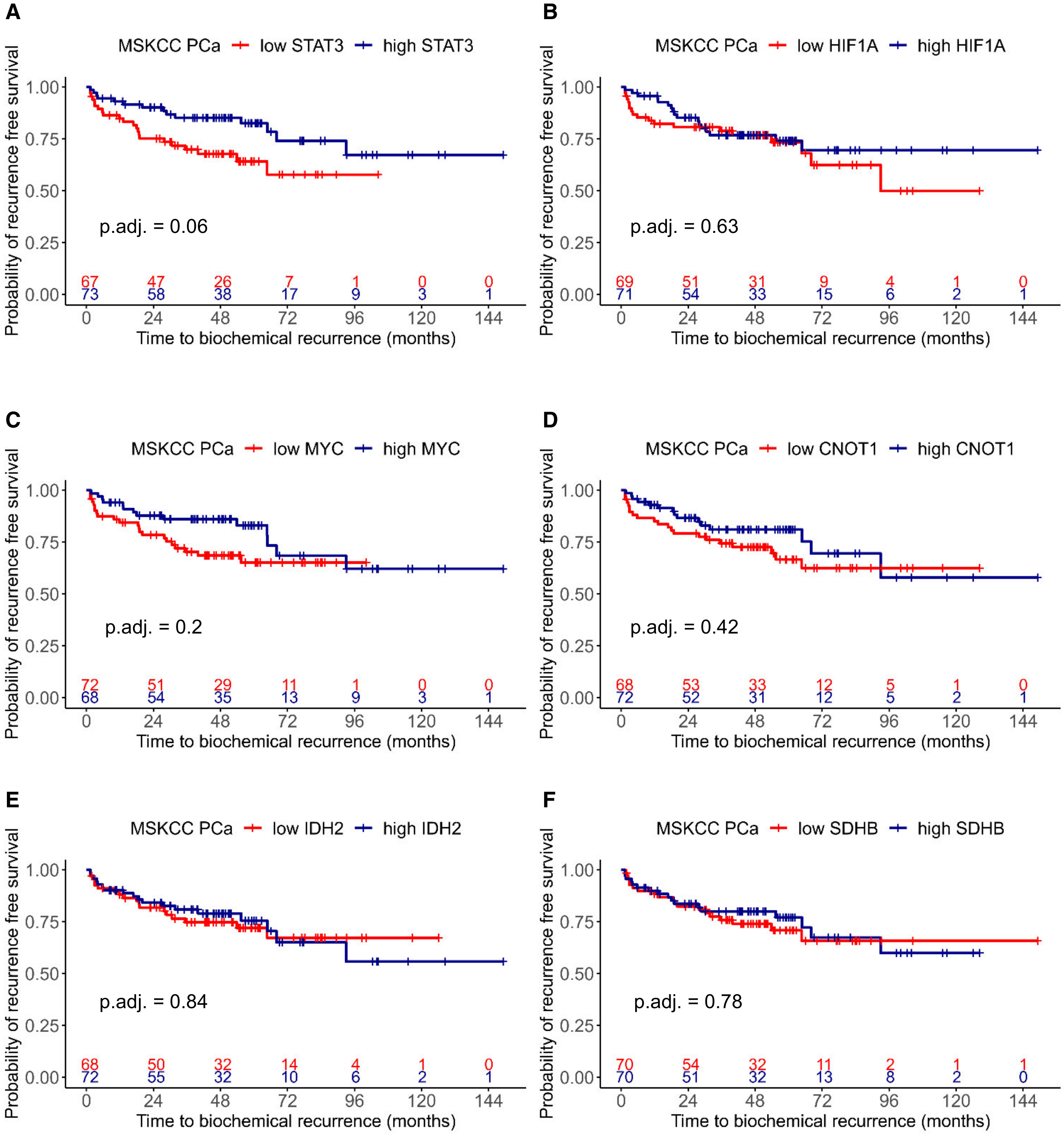

Figure EV4. Kaplan–Meier curves of additional candidate genes.

A–F Time to BCR in months for *STAT3* (A), *HIF-1α* (B), *c-MYC* (C), *CNOT1* (D), *IDH2* (E), and *SDHB* (F) in the MSKCC PCa GSE21032 data set. Groups were generated by a median split. *P*-values were estimated by log-rank test and adjusted with Benjamini–Hochberg method. + = censored.

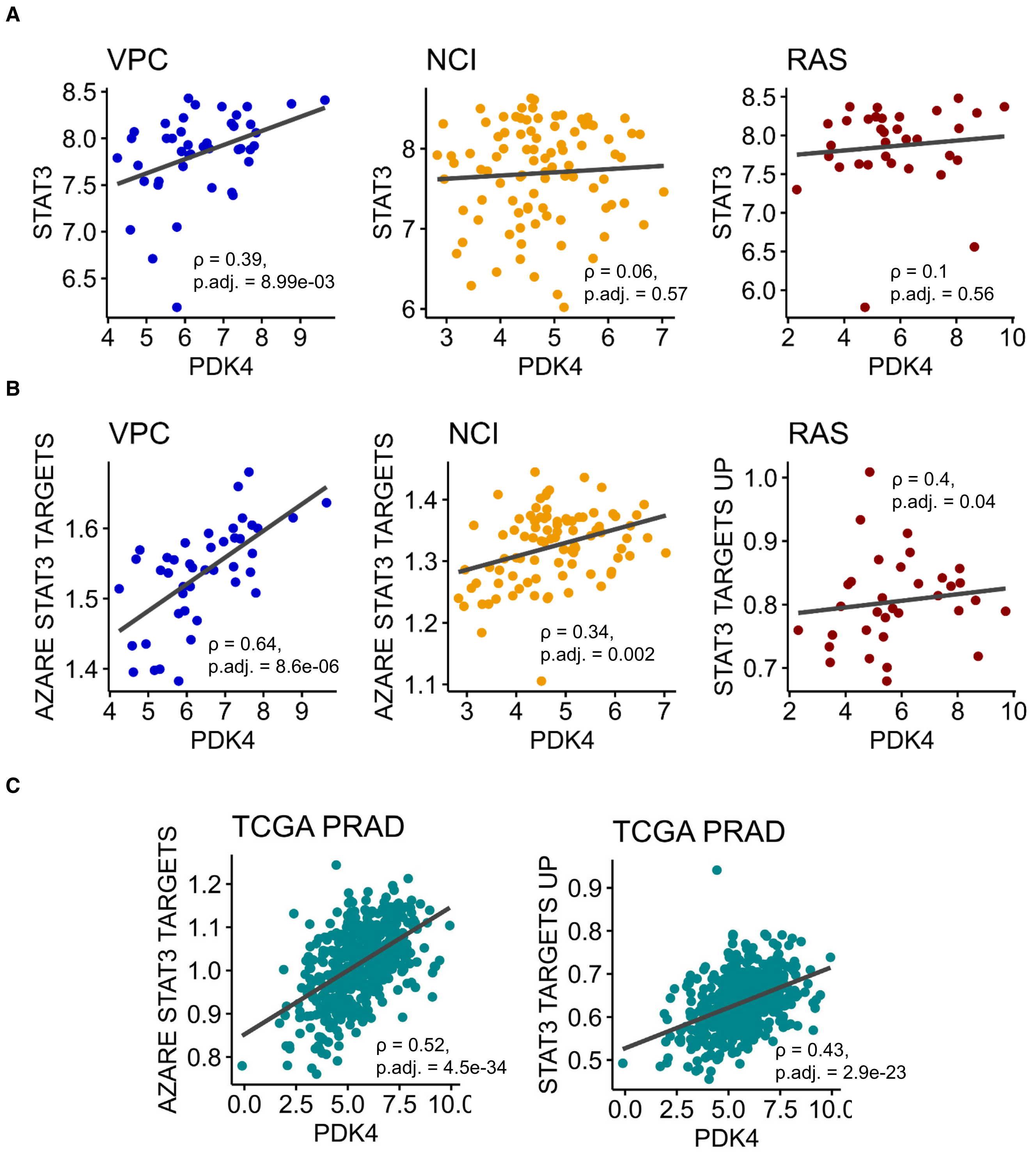

Figure EV5.

© 2020 The Authors

Figure EV5. Correlation of *PDK4* with *STAT3* and STAT3 target signatures.

A Pearson correlation of *PDK4* log counts per million (cpm) with *STAT3* log cpm in three prostate cancer data sets. *P*-values were adjusted with Benjamini–Hochberg method. *P*. adj., adjusted *P*-value. NCI, The Netherlands Cancer Institute; VPC, The Vancouver Prostate Center; RAS, The Russian Academy of Science.

B Pearson correlation of *PDK4* log cpm with "AZARE STAT3 TARGETS" gene signatures in three prostate cancer data sets. Gene signatures were assessed with ssGSEA. *P*-values were adjusted with Benjamini–Hochberg method.

C Pearson correlation of *PDK4* log cpm with "AZARE STAT3 TARGETS" (left) and "STAT3 TARGETS UP" gene signatures (right) in TCGA PRAD. Gene signatures were assessed with ssGSEA. *P*-values were adjusted with Benjamini–Hochberg method. *P*. adj., adjusted *P*-value.

Source data are available online for this figure.

© 2020 The Authors
