# Supplementary figures and images for: STAT3‐dependent analysis reveals PDK4 as independent predictor of recurrence in prostate cancer
Source: Mol Syst Biol. 2020 Apr 23;16(4):e9247. doi: 10.15252/msb.20199247 (PMC7178451; doi:10.15252/msb.20199247)

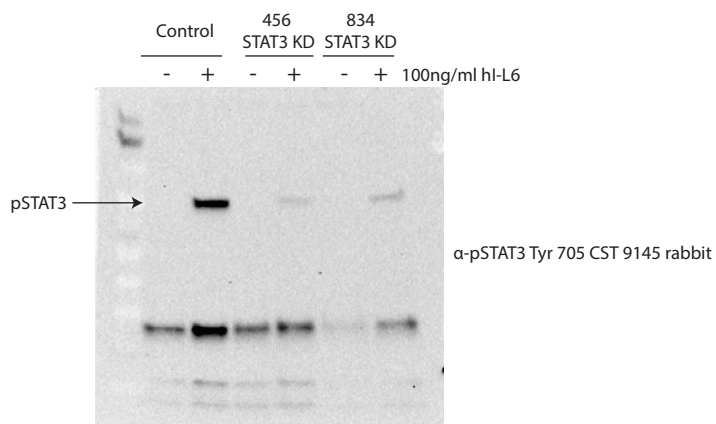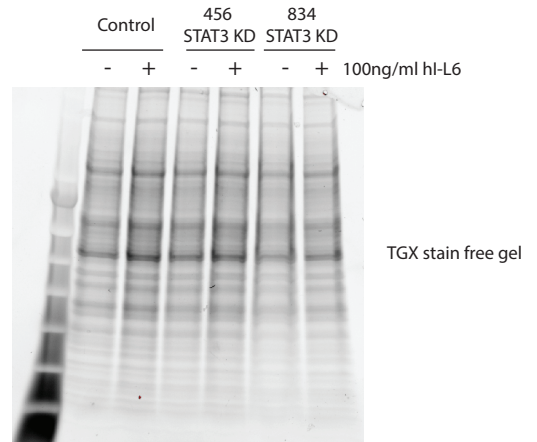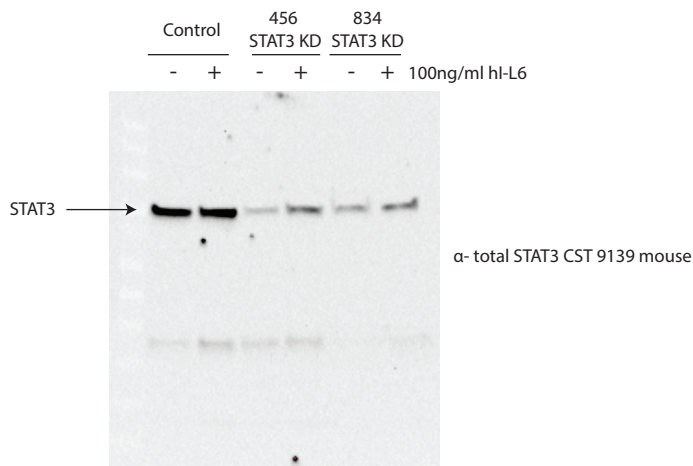

**pSTAT3 normalized to total STAT3 and total protein**

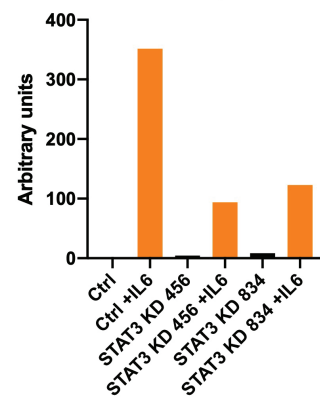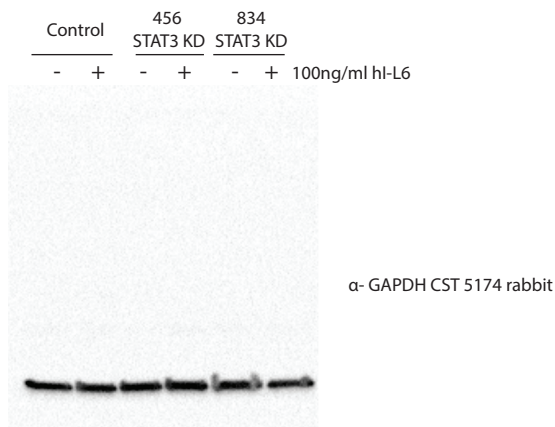

Supplement: Supplementary file 9 — Source Data for Expanded View/Source Data for Expanded View and Appendix [file MSB-16-e9247-s012.zip › MSB-19-9247R_SourceDateforAppendixFigS6C-D/MSB-19-9247R_SourceDateforAppendixFigS6C.pdf]

**Ponceau**

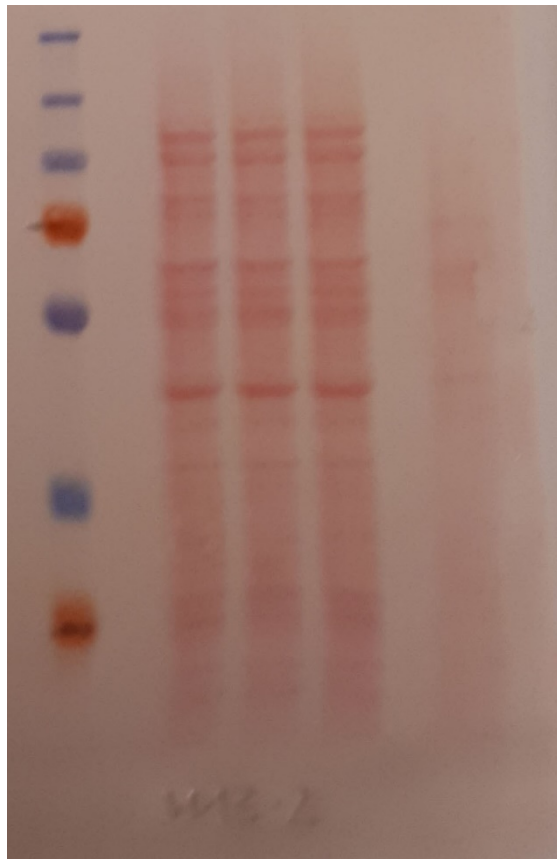

**STAT3**

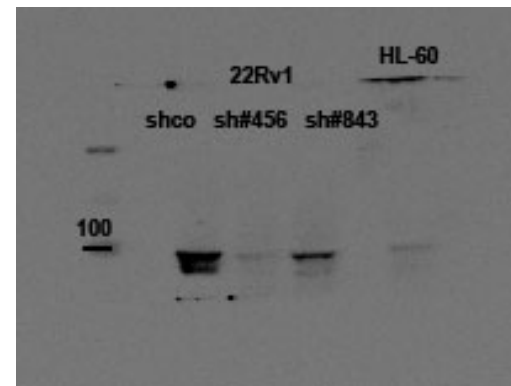

**PDK4**

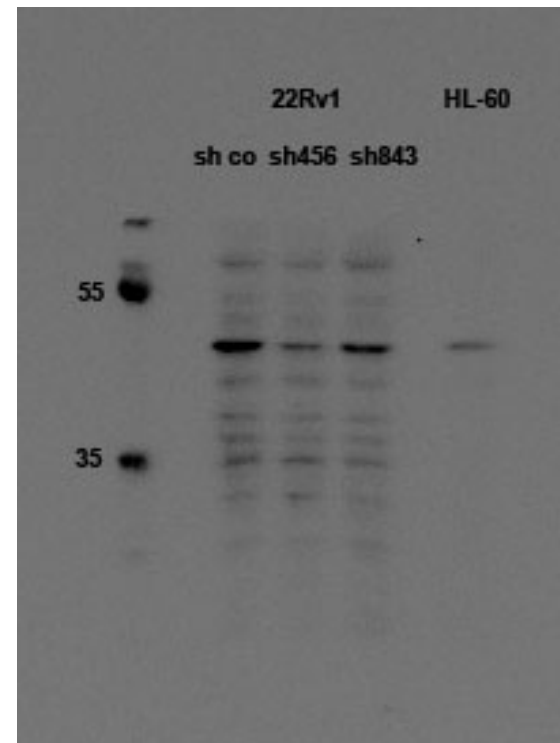

**B-TUBULIN**

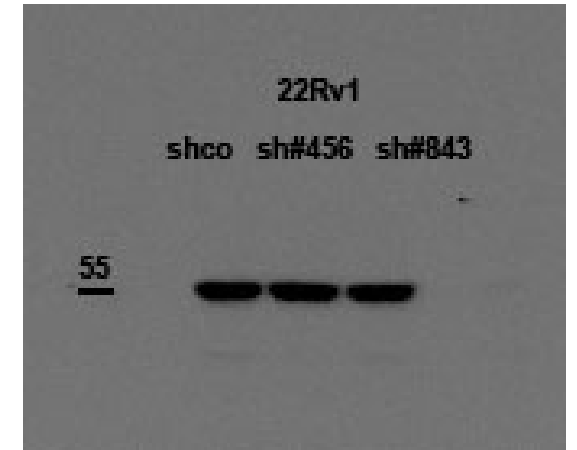

Supplement: Supplementary file 13 — Source Data for Figure 7 [file MSB-16-e9247-s011.pdf]
